# Long Intergenic Non-Coding RNAs (LincRNAs) Identified by RNA-Seq in Breast Cancer

**DOI:** 10.1371/journal.pone.0103270

**Published:** 2014-08-01

**Authors:** Xianfeng Ding, Limin Zhu, Ting Ji, Xiping Zhang, Fengmei Wang, Shaoju Gan, Ming Zhao, Hongjian Yang

**Affiliations:** 1 Institute of Bioengineering, College of Life Science, Zhejiang Sci-Tech University, Hangzhou, Zhejiang, P.R. China; 2 Zhejiang Cancer Research Institute, Department of Breast Tumor Surgery, Zhejiang Cancer Hospital, Banshan Bridge, Hangzhou, Zhejiang, P.R. China; 3 Women's Hospital, School of Medicine, Zhejiang University, Hangzhou, Zhejiang, P.R. China; H. Lee Moffitt Cancer Center & Research Institute, United States of America

## Abstract

In an attempt to find the correlation of aberrant expression of long intergenic noncoding RNAs (lincRNAs) with cancer, twenty-five samples of breast cancer tissue and respective adjacent normal tissue were studied for the expression of lincRNAs by RNA-seq. Among the 538 lincRNAs studied, 124 lincRNAs were exclusively expressed in cancer adjacent tissues and 62 lincRNAs were exclusively expressed in the cancer tissues. Furthermore, the expression of 134 lincRNAs was higher while 272 lower in breast cancer tissue compared with adjacent tissue. The expression of four selected lincRNAs (BC2, BC4, BC5, and BC8) was validated by semi-quantitative and real-time PCR. It was revealed that expression of lincRNA-BC5 was positively correlated with patients' age, pathological stage, and progesterone receptor concentration, while lincRNA-BC8 was negatively correlated with progesterone receptor expression. Higher expression of lincRNA-BC4 was seen in advanced breast cancer grade. LincRNA-BC2 showed no specific changes in the pathological features studied. Interactions between selected lincRNAs and breast cancer associated proteins were highly suggested by *RPIseq* based on the specific secondary structure. The results demonstrated that this group of lincRNAs was aberrantly expressed in breast cancer. They might play important roles in the function of oncogenes or tumor suppressors affecting the development and progression of breast cancer.

## Introduction

The human genome contains only 20,000 protein-coding genes, representing <2% of the total genome. Therefore substantial fractions of the human genome can be transcribed, yielding many short or long noncoding RNAs (ncRNA) with limited protein-coding capacity [1].

Long intergenic ncRNAs (lincRNAs) range in size from several hundred to tens of thousands of bases (≥200). They belong to a newly discovered class of ncRNAs. Although more than 3,000 human lincRNAs have been identified, less than 1% of them have been characterized [2]. Although still largely unexplored, ncRNA, particularly lincRNAs, have emerged as a new regulatory molecule exemplified by their frequent cell-type specific expression and subcellular compartment localization. They may play important roles in numerous systems, and interact with cancer related genes. Indeed, several well-described examples, such as HOTAIR [3], Xist [4], lincRNA-p21 [5], and MALAT-1 [6], indicate that lincRNAs may be essential factors in occurrences and developments of cancer [7], [8].

Breast cancer is now considered a heterogeneous group of diseases with distinct clinical, pathological and molecular features. The latest report on cancer epidemiology indicated that breast cancer was the most common cancer in woman in 2013 [9]. Breast cancer is expected to account for 29% of all new cancer cases among women, and it ranked second in death rates. Recently, noncoding RNA, such as microRNAs [10] and lincRNAs [11], have become a hot topic in the development and progress of breast cancer. However, studies on lincRNAs in breast cancer are at a preliminary stage. Gupta reported that increased expression of HOTAIR was correlated with poor prognosis and tumor metastasis in breast cancer [3]. Recent studies showed that MALAT-1 was up-regulated in many human solid carcinomas, including lung, breast, colon, prostate and HCC [6]. However, the clinical value of lincRNA as an emerging group of ribonucleotides is still largely unknown in breast cancer. Although lincRNAs may have an impact on various human diseases [11], the detailed role and molecular mechanisms are still largely unknown.

Recent advance in RNA sequencing (RNA-seq) and computational methods allowed researchers to comprehensively annotate and characterize lincRNA transcripts [12], [13]. Especially, paired-end RNA-seq, where 36–100 bp were sequenced from both ends of 200 to 500 bp long DNA molecules, was suitable for the detection of low-copy and novel lincRNAs [14].

To better understand the roles of lincRNAs in breast cancer development and progression, comprehensive analysis of the expression abundance of lincRNAs is required. In this study, we described a comprehensive analysis of lincRNAs in twenty five pairs of snap-frozen breast cancer tissues and matched cancer adjacent tissues by RNA-Seq. We found that a group of lincRNAs was aberrantly expressed in twenty five breast cancer tissues compared with matched adjacent tissues. Five samples randomly chosen from these patients were analyzed for the expression of lincRNAs, by deep sequencing technology. Twenty samples from breast cancer patients were evaluated by real-time qPCR. The correlation between the expression levels of the selected lincRNAs with pathological parameters was analyzed. In order to further study the functional mechanism, secondary structures of the lincRNAs were projected by software *RNAfold* Web Server. Meanwhile, the possibility of interaction between lincRNAs and oncogenes was evaluated by *RPIseq*.

## Results

### RNA-Seq and Reads mapping

PolyA-minus RNAs were fractionated from total RNA samples isolated from pooled breast cancer tissues and matched adjacent tissues. Then, RNA-seq libraries were generated by RNA-fragmentation, random hexamer-primed cDNA synthesis, linker ligation and PCR amplification. The purified DNA libraries were sequenced by using Illumina Hi-seq 2000 platform.

**Figure 1:**
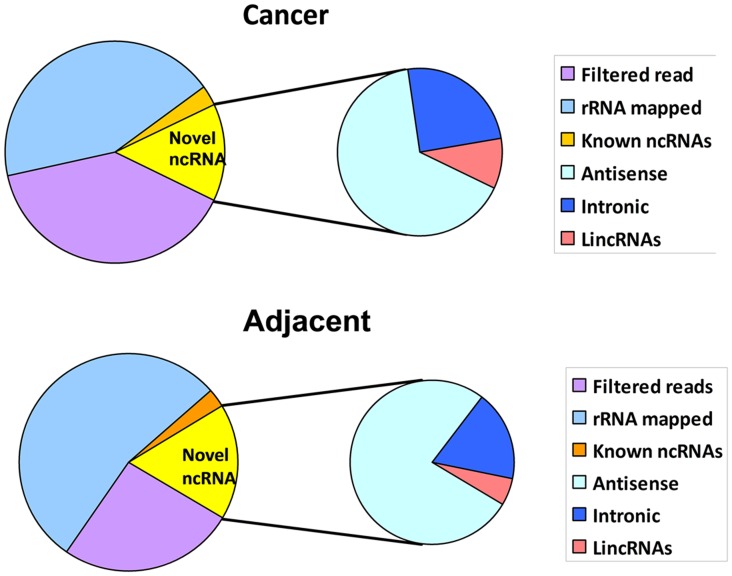
Global overview of polyA-minus RNA sequencing in breast cancer tissues and matched adjacent cancer tissues. The pie charts on the left display ploy-A minus transcript distribution in breast cancer tissues (upper) and adjacent tissues to cancer (lower). The pie charts on the right display novel ncRNA categorized as sense (intronic RNA and lincRNA) and antisense transcript.

A total of 27 million reads were obtained. 8.9 million-reads were from cancer tissues, and 18.1 million from the adjacent tissues (Figure 1). The obtained reads were first mapped to the human reference genome using the TopHat program (v1.0.3). Reads mapped to genome were then filtered against the RepeatMask and Ensembl gene sets, which resulted in the identification of novel intergenic transcription regions. These reads were then compared against rRNA and other repeated sequences. Finally there were 5.41 million and 13.4 million reads in breast cancer tissues and adjacent tissues, respectively after depletion of rRNA database matching with BOWTIE (0.12.7). 17.9% and 13.07% were mapped to the NONCODE 2.0 using TopHat (1.2.0) in cancer tissues and adjacent tissues respectively (Figure 1).

### Bioinformatics analysis

For each transcription region, a FPKM (fragment per kilobase of transcript per million mapped reads) value was calculated to quantify its expression abundance and variations, using cufflinks v1.0.3.

To accurately define the boundaries of these intergenic transcript regions, two parameters were considered: the minimum distance from the two neighboring genes and the minimum number of mapped reads within a genomic region. The minimum distance was set 1500 nucleotides after the terminal of the upstream gene and also 1500 nucleotides before the origin of downstream gene. 10 mapped reads were considered as the standard minimum number. Based on these two principles, 538 long intergenic noncoding RNAs (lincRNAs) were identified with consistency (Table S9). Among them, there were 124 lincRNAs (Table S1) exclusively expressed in the tissues adjacent to cancer (FPKM>10) and 62 lincRNAs (Table S2) exclusively expressed in the cancer tissues (FPKM>10). Furthermore, 352 overlapped lincRNAs in both cancer and adjacent tissues were aberrantly expressed through calculating the fold-change of RPKM (the absolute value by log2 (reads in cancer tissues/reads in adjacent tissues to cancer)≥1). 134 lincRNAs were expressed higher (Table S3) and 272 lincRNAs (Table S4) were expressed lower in breast cancer tissues compared with adjacent breast cancer tissues. Considering their diverse functions in carcinogenesis, several most different lincRNAs from both up-regulated subgroup and down-regulated subgroup were selected to be further studied. This selection method was based on four principles. Firstly, the selected lincRNA were stability expressed in all the samples we ever used in our study. Secondly, the results of semi-quantitation PCR preliminary indicated the differences between breast cancer tissues and adjacent tissues. Thirdly, forming stable scaffold structure and having entropy for the lincRNA are two important factors. Fourthly, the clear locations of lincRNAs and gene on both sides are considered to screening lincRNAs for further study. The selected lincRNAs were named by the abbreviation of breast cancer (BC), such as lincRNA-BC2 (log2 Ratio = 2.46) and lincRNA-BC5 (log2 Ratio = 2.22) were the most significantly up-regulated lincRNAs, and lincRNA-BC4 (log2 Ratio = −2.64) and LincRNA-BC8 (log2 Ratio = −2.017) were the most significantly down-regulated ones.

### Individual validation of lincRNAs by RT-PCR and qPCR

**Figure 2:**
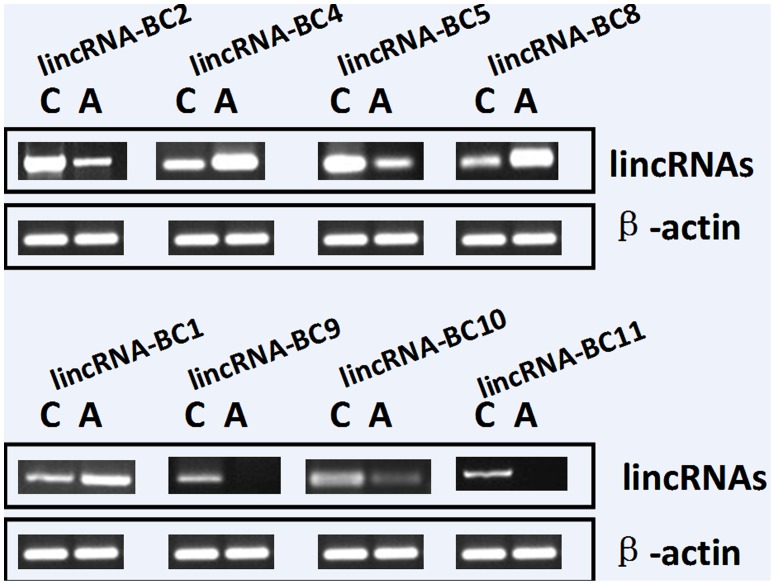
The expression of selected lincRNAs in breast cancer tissues and matched adjacent tissues to cancer using semi-quantitative RT-PCR.

The solexa results from two pooled samples of five breast cancer tissues and matched adjacent cancer tissues were further validated individually by semi quantitative and real-time PCR. There were twenty (all the numbers are confusing and did not make sense in the current description) breast cancer tissues and matched adjacent tissues used in these experiments. The expression of these selected lincRNAs was validated by using RT-PCR and qPCR (Figure 2). Selected lincRNAs (Table S5) were validated by RT-PCR and the information of primers was provided in Table S6.

### LincRNAs expression and clinical pathologic feature of breast tissues

**Figure 3:**
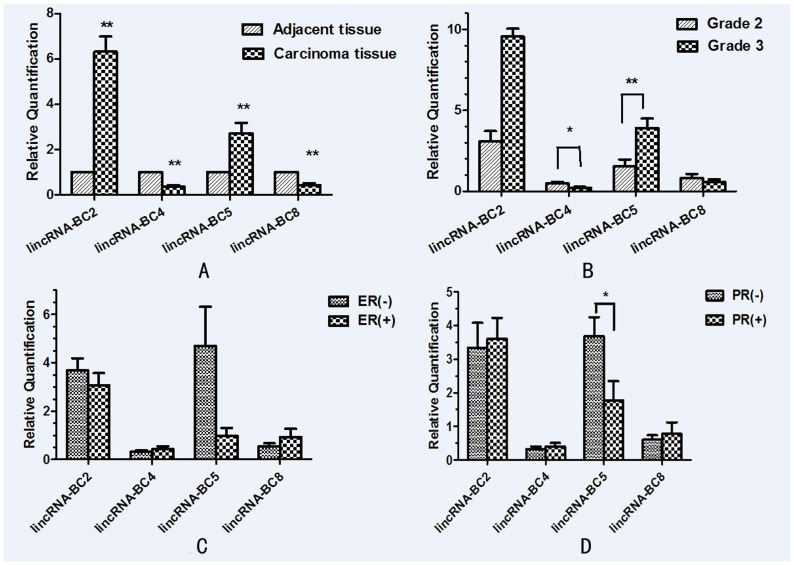
Comparison of lincRNAs (BC2, BC4, BC5, BC8) aberrant expression in breast cancer tissues with matched adjacent tissues to cancer with clinic pathological. (A) Comparison of lincRNA aberrant expression in breast cancer tissues with matched adjacent tissues to cancer. (B) Comparison of lincRNA aberrant expression in breast cancer tissues on tumor grades between grade II and grade III. (C) Comparison of lincRNA aberrant expression in breast cancer tissues on the level of ER (estrogen receptor). (D) Comparison of lincRNA aberrant expression in breast cancer tissues on the level of PR (progesterone receptor).

The expression of selected lincRNAs was analyzed in 20 breast cancer tissues and matched adjacent tissues. We analyzed lincRNAs whose expression was significantly different between cancer tissues and adjacent tissues. LincRNA-BC2 (6.315±0.672, P = 0.00) and lincRNA-BC5 (2.72±0.46, P = 0.001) were consistently up-regulated more than 2-fold (mean±SD) in cancer samples. Whereas, lincRNA-BC4 (0.358±0.062, P = 0.00) and lincRNA-BC8 (0.436±0.0732, P = 0.00) were down-regulated (p<0.01) (Figure 3A.). The consistent differential expression suggested that they could potentially act as tumor oncogenes or suppressor genes, respectively. We also analyzed the correlation between lincRNA expression and the clinical feature of the cancer including the ages of original diagnosis (median 54 years, range 32–73 years), cancer staging (grade II or III), lymph node metastasis (positive or negative), ER/PR (estrogen and progesterone receptor level), the level of HER2 (positive or negative) and p53 protein (positive or negative).

Expression of LincRNA-BC4 (P = 0.03) was significantly lower in grade III breast cancers compared to that in grade II cancers. On the contrary, the expression of lincRNA-BC5 (P = 0.007) was significantly higher in grade III. Meanwhile, there were no significant differences of lincRNA-BC2 (P = 0.301) and lincRNA-BC8 (P = 0.441) on the different grades in pathology (Figure 3B).

The expression of the lincRNAs (BC2, BC4, BC8) did not exhibit significant differences between ER positive (ER+) patients and ER negative(ER-)patients (P = 0.502, 0.438 and 0.344 respectively). Only lincRNA-BC5 (P = 0.00) expression was significantly lower in estrogen receptor negative patients compared to positive patients (Figure 3C). LincRNA-BC5 (P = 0.031) expression was also significantly lower on the progesterone receptor (PR) negative patients. The expression of lincRNAs BC2, BC4, and BC8 exhibited no difference between PR negative and PR positive (P = 0.782, 0.568 and 0.642 respectively) (Figure 3D).

**Figure 4:**
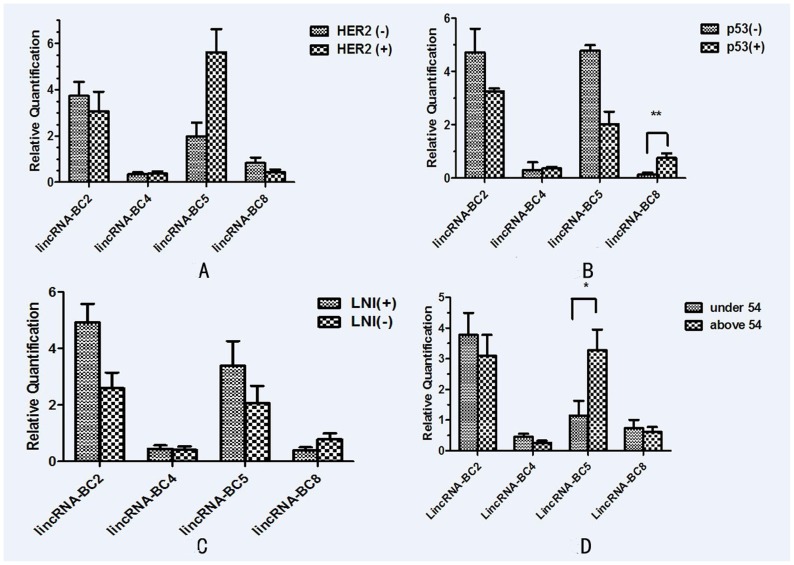
Comparison of lincRNA aberrant expression in breast cancer tissues with matched adjacent tissues to cancer in clinic pathological. (A) Comparison of lincRNA aberrant expression in breast cancer tissues with matched adjacent tissues to cancer on the level of HER-2. (B) Comparison of lincRNA aberrant expression in breast cancer tissues on the level of p53. (C) Comparison of lincRNA aberrant expression in breast cancer tissues on the level of LNI (lymph node metastasis). (D) Comparison of lincRNA aberrant expression in breast cancer tissues on the age of patients (<54 and ≥54).

The expression of lincRNAs were not significantly different between HER2-negative and HER2-positive breast cancer tissues (for lincRNA-BC2, P = 0.542; for lincRNA-BC4, P = 0.866; for lincRNA-BC5, P = 0.176; lincRNA-BC8, P = 0.166) (Figure 4A). However, lincRNA-BC8 (P = 0.004) showed significantly higher expression in the p53-positive cancer tissues compared to the p53-negative ones. LincRNA-BC2 (P = 0.526), lincRNA-BC4 (P = 0.867), and lincRNA-BC5 (P = 0.393) were not significantly different between p53 positive and p53 negative breast cancer tissues (Figure 4B).

The expression of lincRNA-BC2 (P = 0.017) was significantly higher in patients with lymph node metastasis compared to patients without metastasis. The expression of other lincRNAs, BC4, BC5 and BC8 were not significantly different between the metastatic and non-metastatic patients. In terms of age, lincRNA-BC5 (P = 0.021) was little expressed in older group (>54 years) compared to younger group (≤54 years). LincRNA-BC2(P = 0.502), lincRNA-BC4(P = 0.438), and lincRNA-BC8(P = 0.344) did not show statistically significant difference between the two groups. (Figure 4D). The information of qPCR primers was provided in Table S7, and the average levels of lincRNAs expression and correlation with clinic pathology was presented in Table S8.

The qPCR result showed that there was a significant correlation between lincRNAs and carcinogenesis. LincRNA-BC5 was specially related with high degree of differentiation and elder age. However, LincRNA-BC2 was a factor unrelated to hormone level, but more highly expressed in breast cancer. It was suggested that lincRNA-BC2 might play a role in triple negative breast cancer.

### Validation of chromosomal location

**Figure 5:**
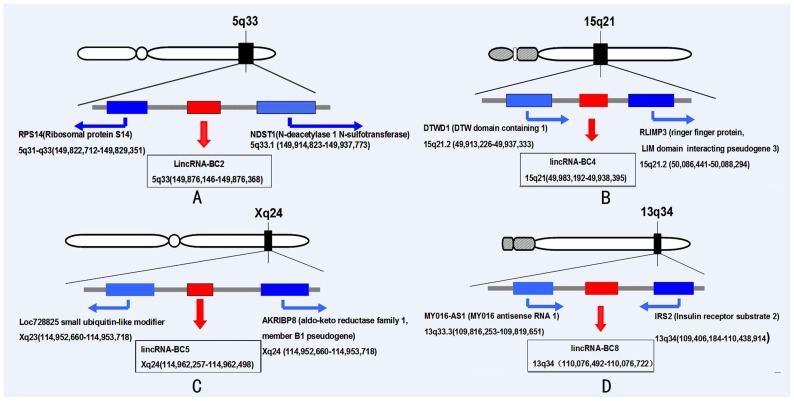
The position of lincRNAs and neighboring genes in chromosome. (A) The position of lincRNA-BC2 and neighboring genes in chromosome 5. (B) The position of lincRNA-BC4 and neighboring genes in chromosome 15. (C) The position of lincRNA-BC5 and neighboring genes in chromosome X. (D) The position of lincRNA-BC8 and neighboring genes in chromosome 13.

In order to prove the accuracy of the selected lincRNAs, the “intergenic” positions need to be validated precisely. The transcription initiation sites and termination sites of bilateral genes and lincRNAs were exactly mapped on chromosomes. We validated the position of the four lincRNAs on chromosomes through BLASTN alignments from NCBI Genebank. Each “intergenic” region and adjacent gene was confirmed. For lincRNA-BC2 (chr5q33: 149,876,146–149,876,368), the upstream gene was RPS14 (40S ribosomal protein S14), and the downstream gene was NDST1 (N-deacetylase/N-sulfotransferase (heparin glucosaminyl). LincRNA-BC4 (chr15q21: 49,983,192–49,938,395) was between DTWD1 (DTW domain containing 1) and RLIMP3 (ringer finger protein, LIM domain interaction pseudogene 3). LincRNA-BC5 (chrXq24: 114,962,257–114,962) was between Loc728825 small ubiquitin-like modifier and AKRIBP8 (aldo-keto reductase family 1 member B1 pseudogene). For lincRNA-BC8 (chr13q34: 110,076,492–110,076,722), the bilateral genes were MY016-AS1 (MY016 antisense RNA1) and IRS2 (Insulin receptor substrate 2). The loci information was consistent with the result from bio-informatics analysis. The transcriptional initiation sites and termination sites of neighbor genes on chromosome were shown in Figure 5.

### Prediction of LincRNAs' secondary structure

RNA sequences are single-strand biopolymers which can fold themselves. The potential interactions in organism are determined by RNA secondary structure [15]. The prediction of RNA structure can be the first important step for the functional characteristics of novel lincRNAs [16]. The structure of the selected lincRNA were predicted with computer software *RNAfold* Web Server (http://rna.tbi.univie.ac.at/cgi-bin/RNAfold.cgi).

**Figure 6:**
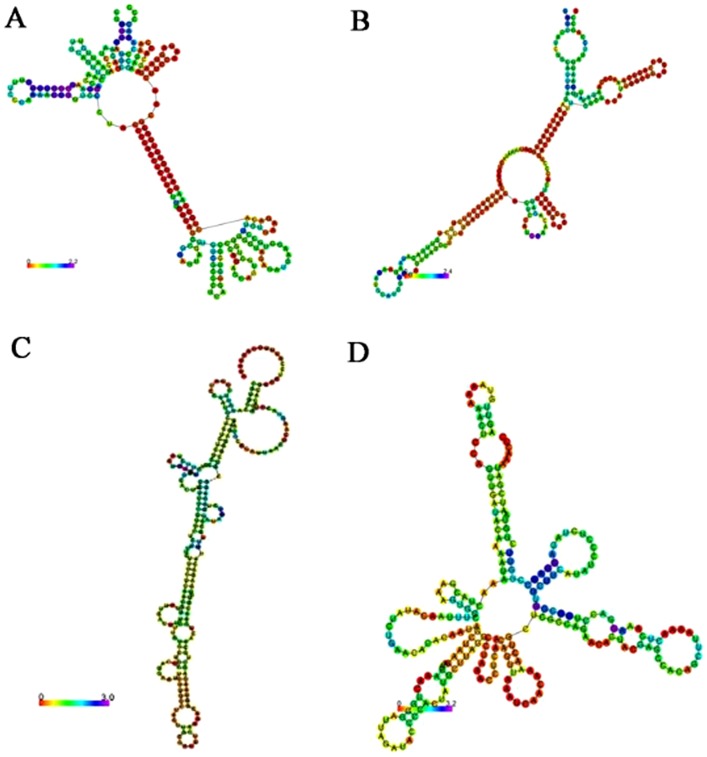
The second structure predicted by *RNAfold*. (A) The second structure of lincRNA-BC2 predicted by *RNAfold*. (B) The second structure of lincRNA-BC2 predicted by *RNAfold*. (C) The second structure of lincRNA-BC2 predicted by *RNAfold*. (D) The second structure of lincRNA-BC2 predicted by *RNAfold*.

The results showed that they all had low free energy from algorithms. Meanwhile, there were more than three stem-loop structures by self-fold (Figure 6). This specific structure means that there probably were proteins or chromosomes binding sites. It was suggested that the selected lincRNAs may be involved in the complex chromatin-modifying complexes or in the regulation of gene transcription.

### *RPISeq* prediction

RNA-protein interactions play important roles in a wide variety of cellular processes, ranging from transcriptional and post-transcriptional regulation of gene expression to host defenses against pathogens [17]. RNA-Protein interaction prediction was performed by *RPIseq*. *RPISeq* (http://pridb.gdcb.iastate.edu/RPISeq/), a family of purely sequence-based classifiers, can be used to predict whether a specific RNA-protein is likely to interact. Two variants of *RPISeq* were presented:*RPISeq-SVM* (Support Vector Machine (SVM) classifier) and *RPISeq-RF* (Random Forest classifier). Predictions with probabilities >0.5 were considered positive.

**Figure 7:**
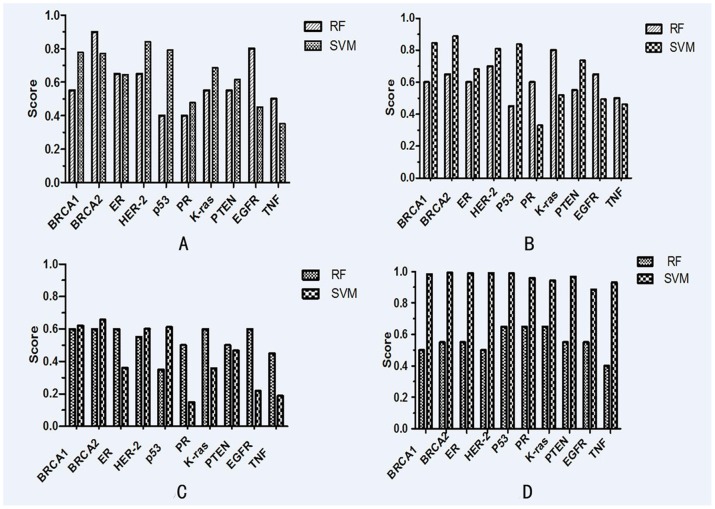
The scores of the interaction probability between lincRNAs and breast cancer associated protein predicted by *RPIseq*. (A) The scores of the interaction probability between lincRNA-BC2 and breast cancer associated protein predicted by *RPIseq*. (B) The scores of the interaction probability between lincRNA-BC4 and breast cancer associated protein predicted by *RPIseq*. (C) The scores of the interaction probability between lincRNA-BC5 and breast cancer associated protein predicted by *RPIseq*. (D) The scores of the interaction probability between lincRNA-BC8 and breast cancer associated protein predicted by *RPIseq*.

The results showed that all four lincRNAs had great possibility of interaction with BRCA1 and BRCA2 (Figure 7). For example, LincRNA-BC2 was predicted to interact with BRCA1 by RF (P = 0.55) and SVM (P = 0.779). It was also predicted to interact with BRCA2 by RF (P = 0.90) and SVM (P = 0.772). All of the four lincRNAs were predicted to interact with ER or HER2 by scores of RF and SVM bigger than 0.5. It was therefore suggested that these four lincRNAs may involve in the occurrence of breast cancer since ER and HER2 had been used as clinical biomarkers for diagnosis. The other proteins targeted for potential interaction with the lincRNAs were listed in the Figure 6. However, there were different results of prediction for interaction of lincRNA-BC8 with proteins by RF classifier (0.4–0.65) and SVM classifier (0.886–0.989).

## Discussion

The development of high throughput deep sequencing technology provided the possibility of a nearly complete view of lincRNAs profiles [18]. Deep sequencing technology had the potential to identify novel tissue-specific lincRNAs. This new technology had the advantages of providing not only sequence of low abundance species, but also quantitative data since the frequency of sequencing reads reflects the abundance of lincRNAs in the population.

Recent studies demonstrated that lincRNAs are exquisitely regulated during the development of cancer. They responded to diverse signaling cues and were aberrantly expressed in diverse cancers tissues.

In this study, deep sequencing technology was used to detect the expression profiles of lincRNA in five pairs of snap-frozen breast cancer tissues and matched adjacent cancer tissues. Expression of many lincRNAs was significantly altered in cancer tissues compared with matched adjacent tissues, suggesting that these aberrantly expressed lincRNAs might play roles in carcinogenesis. Real-time qPCR was performed to evaluate the expression pattern of lincRNA-BC2, lincRNA-BC4, lincRNA-BC5, and lincRNA-BC8 in twenty carcinoma patients. LincRNA-BC2 and lincRNA-BC5 expression were upregulated while; lincRNA-BC4 and lincRNA-BC8 expression were down-regulated in breast cancer tissues compared to matched adjacent tissues. These findings were consistent with the results from deep sequencing analysis.

We used the matched adjacent tissues as control against the cancer tissue in our study. However, it was previously reported that preliminary changes on transcription took place in tissues adjacent to the carcinoma. Due to the ethical difficulties of obtaining normal breast tissues, β-actin housekeeping gene was used as internal control gene in the validation phase [19]. Expression of β-actin was relatively constant in both breast cancer tissues and adjacent tissues. Therefore β-actin could be used as a control normalizer in real-time quantitative PCR.

The function of the selected lincRNAs (lincRNA-BC2, BC4, BC5, BC6) remain largely unknown but the result demonstrated that they were aberrantly expressed in cancer tissues compared to adjacent cancer tissues by qPCR. Especially the expression level of the lincRNA-BC5 was positively correlated with patients' age, pathological stage, and progesterone level which were statistically significant.

The locations of the four studied lincRNAs were also extremely important. For the position of lincRNA-BC2, Dhillon et al. indicated that chr5q33 as a fragile site may be the unstable sites in the genome and can be used as suitable and reliable markers for genetic factor to breast cancer, epithelial ovarian cancer, and in non-small-cell lung cancer [20]. LincRNA-BC4 was mapped to chr15q21. Recent studies provided increasing evidence that chromosomal arm 15q may be an important target of genetic alterations in the progression of breast cancer [21]. The expression of chr15q21 was proved to be related with the risk of Griscelli syndrome [22], chronic lymphocytic leukemia [23], and prostate cancer. LincRNA-BC5 was located on chromosome Xq24. This region was reported to be associated with height [24]. In addition, the transcription level of this region might influence the risk level of obesity [25]. LincRNA-BC8 was mapped to Chr13q34 which was quite an interesting location. Lorenzo et al [26] reported that 13q34 amplification was a genomic aberration, and it was associated with basal-like breast cancer. Furthermore, this amplification had been previously reported in squamous cell carcinomas [27], adrenocortical carcinomas [28], childhood medulloblastoma [29], hepatocellular carcinomas [30] and breast cancer [26]. These lincRNAs selected from the solexa data of breast cancer tissues were mapped to disease-associated loci. Further studies are required to discover their important roles in carcinogenesis.

LincRNAs target chromatin modification complexes or RNA-binding protein to alter gene expression programs. One of the well-characterized lincRNAs is HOTAIR, which is transcribed between the HOXC clusters and represses genes in the HOXD cluster by binding and recruiting the chromatin-modifying complex PRC2. LincRNAs may carry out lots of functions by acting as modular scaffolds for protein-chromatin interactions [31]. Tsai et al. suggested that lincRNAs may serve as scaffold by providing binding surfaces to assemble selected histone modification enzymes, thereby specifying the pattern of histone modification on target genes. LincRNAs play multiple functions with specific structure of several stem loops. In this study, the presence of multiple binding sites enables the RNA to specifically associate with DNA, RNA, and/or protein.

The precise mechanism of lincRNAs function remains poorly understood. However, one emerging theme is the interaction between lincRNAs and proteins. The functional importance of many lincRNA-protein interactions in transcriptional regulation has been demonstrated, such as XIST,HOTAIR and lincRNA-p21. *RPIseq* was used to predict the interaction between lincRNAs (BC2, BC4, BC5 and BC8) and breast cancer associated protein. *RPIseq* was a reliable method using only sequence-derived information [17]. Davide et al. had used this method to study the probabilities of interactions between HOTAIR and Suz12 [32].

We analyzed the interaction between lincRNAs and breast cancer associated proteins including BRCA-1 [33], BRCA-2 [34], PR, ER [35], p53, HER2 [36], K-ras [37], PTEN [38], TNF [39], and EGRF. All of the ten proteins were identified to play important roles in development, especially in the progress of tumor invasion and metastasis [40]. *RPIseq* is a sequence-based predictive method. The accuracy of the prediction ranged from 57–99% [17] in independent datasets of RNA-protein interactions. We found that most of scores were more than 0.5 analyzing interaction probabilities between the four lincRNAs and the proteins. It suggested that the four lincRNAs may participate in the carcinogenesis. However, computational prediction of lincRNA functions is still at its primary stage. More softwares were required to confirm the potential interactions of lincRNA. In addition, experimental methods such as RNA Knocking-Out, Western Blotting should be adopted in the future research.

Our data provides novel insight into breast cancer biology. A collection of lincRNAs was aberrantly expressed in breast cancer, suggesting that they might play roles as oncogenes or tumor suppressors in the development and progression of cancer. But it is necessary that large amount of breast cancer samples need to be collected to verify our results. Therefore, more work will be done to determine potential functions and regulatory mechanism of lincRNAs in breast cancer.

## Materials and Methods

### Ethics statement

Tissues from twenty-five women with breast cancer were collected from Zhejiang Cancer Hospital (time from February of 2008 to December of 2010). This study was approved by Zhejiang Provincial Experimental Animal Manage Committee under Contract 2013–2069 (ZEAC 2013–2069). All aspects of the study comply with the Declaration of Helsinki. All the samples were collected with informed consent of the patients. Ethics Committee specifically approved that not informed consent was required because data were going to be analyzed anonymously. Furthermore, there is no security and privacy violation to the patient's health in our study.

### Preparation of patients' samples

None of the patients had received any radiotherapy and/or chemotherapy. All the patients were diagnosed as infiltrating carcinoma by pathology. Fresh tissues were harvested from patients, snap-frozen, and preserved at −80°C until further use. Clinical and pathological parameters of patients were recorded once diagnosed with breast cancer, including the value of ER, PR, HER-2. p53 and the age of patients. Patient characteristics were summarized in Table 1. Twenty-five breast cancer tissues and their corresponding adjacent tissues to cancer from patients were collected. Of these patients, five were used for initial deep sequencing analysis of lincRNAs and twenty were used for validation by qPCR.

**Table 1:** Clinic pathologic characteristics of patients with breast cancer.

Variable	Clinic pathologic parameter	Number of cases	Number of cases for qPCR
Case		25	20
Age	≤54	13	10
	>54	12	10
Therapy	no	25	20
	Chemotherapy	0	0
	Radiotherapy	0	0
Histological grade a	I	0	0
	II	10	10
	III	15	10
ER	negative	14	13
	positive	11	7
PR	negative	15	12
	positive	10	8
p53	negative	3	3
	positive	22	17
HER2	negative	15	13
	positive	10	7
Lymph node metastasis	no	11	13
	1	14	0
	≥1	0	7
	unknown	0	0

a According to the AJCC (American Joint Committee on Cancer) staging system [42].

### PolyA-minus RNA preparation and next generation sequencing

Total RNA was isolated from breast cancer tissues and adjacent tissues using miRNeasy Kit according to the manufacturer's instructions. An additional DNase I digestion step was performed to ensure that the samples were not contaminated with genomic DNA. RNA purity was assessed using the Nanodrop-2000. Each RNA sample had an A260:A280 ratio above 1.8 and A260:A230 ratio above 2.2.

Five µg of breast cancer tissues were subjected to ribosomal RNA depletion according to the manufacturer's protocol of Ribominus kit. Then RNA was fragmented into ∼200 base pairs (bp) and quantified with Nanodrop. The cDNA libraries were generated by RNA fragmentation, random hexamer-primed cDNA synthesis, linker ligation and PCR amplification.

cDNA was then used for Illumina sequencing library preparation. DNA fragment (200 ng) was then end-repaired to generate blunt ends with 5′ phosphatase and 3′ hydroxyls and adapters were ligated for paired end sequencing on Illumina HiSeq 2000. Purified cDNA library products were then evaluated using the Agilent bio analyzer and diluted to 10 nM for cluster generation in situ on the HiSeq paired-end flow cell using the CBot automated cluster generation system followed by massively-parallel sequencing (2×100 bp) on HiSeq 2000. We obtained 101 bp mate-paired reads from DNA fragments of an average length of 250 bp (standard deviation for the distribution of inner distances between mates pairs is approximately 50 bp). RNA-seq reads from cancer tissues and adjacent tissues were separately aligned to the human genome using the software TopHat (version.1.1.4).

### Quantitative RT-PCR

The clinical characteristics of the breast cancer patients have been described in Table 1. Real-time quantitative RT-PCR was performed to determine gene expression in the samples. Total RNA was isolated using the Qiagen RNeasy kit. First strand cDNA was synthesized as the following: total of 1 µg of RNA from each sample was reverse-transcribed using random primers and M-MLV reverse transcriptase according to the protocol of the manufacturer. Quantitative PCR was performed using SYBR Premix Ex Taq. qPCR was done in triplicates in the ABI prism 7300 sequence detector. The relative amounts of gene expression were calculated (ΔΔCT method) by using the expression of β-actin as an internal standard. The formula based on the threshold cycle (Ct) [41] is as follows:
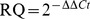



Here, ΔΔCt = (Ct lincRNA- Ct β-actin)cancer–(Ct lincRNA- Ct β-actin)adjacent.

### General statistical analysis for qPCR

Real time qPCR was repeated at least in three independent experiments in every sample. Data were presented as mean±SD of three or more independent experiments. Statistical analysis was performed with SPSS (version17.0) and the differences were considered statistically significant when P value was less than 0.05 by using the independent samples t-test.

## Supplementary Information

The result of the next sequencing was shown in Table S1, S2, S3, S4, and S9. The information of selected lincRNAs for validated was shown in the Table S5. The information of primers for RT-PCR and qPCR was shown in the Table S6 and Table S7. The result of correlations analysis was shown in the Table S8.

## Supporting Information

Table S1 **The 124 lincRNAs exclusively expressed in adjacent tissues to cancer by deep sequencing.**
(XLS)

Table S2 **The 62 lincRNAs exclusively expressed in the cancer tissues.**
(XLS)

Table S3 **The up-expressed lincRNAs in breast cancer tissues compared with adjacent tissues.**
(XLS)

Table S4 **The down-expressed lincRNAs in breast cancer tissues compared with adjacent tissues.**
(XLS)

Table S5 **The information of selected lincRNAs for semi-quantitative PCR.**
(XLS)

Table S6 **The primers information of lincRNAs for RT-PCR.**
(XLS)

Table S7 **The primers information of lincRNA for qPCR.**
(XLS)

Table S8 **The results of data from real-time PCR in breast tissues analyzed by RQ and SPSS 17.0.**
(XLS)

Table S9 **The total aberrantly expression lincRNAs in breast cancer tissues by deep sequencing.**
(XLS)
